# Combined effects of loneliness and inflammation on depression in people with HIV

**DOI:** 10.1007/s13365-023-01145-z

**Published:** 2023-08-31

**Authors:** Mariam A. Hussain, C. Wei-Ming Watson, Erin E. Morgan, Robert K. Heaton, Scott L. Letendre, Dilip V. Jeste, David J. Moore, Jennifer E. Iudicello

**Affiliations:** 1 San Diego State University, University of California San Diego Joint Doctoral Program in Clinical Psychology, San Diego, USA; 2 Department of Psychiatry, University of California San Diego, La Jolla, USA; 3 HIV Neurobehavioral Research Program, University of California San Diego, San Diego, USA; 4 Department of Neurosciences, University of California San Diego, La Jolla, USA; 5 Sam and Rose Stein Institute for Research On Aging, University of California San Diego, La Jolla, USA

**Keywords:** (4–6): Social determinants of health, Psychological distress, Coagulation, D-dimer, Monocyte chemoattractant protein-1

## Abstract

**Objective:**

Loneliness is prevalent in people with HIV (PWH) and associated with adverse health-related consequences, including depression. Chronic inflammation has been linked to depression in PWH, though its association with loneliness is less well established. Simultaneous examination of inflammation, loneliness and depression is needed to clarify these relationships. This study investigated the relationship between loneliness and inflammation, and the effects of loneliness and inflammation on depression in PWH.

**Methods:**

82 PWH who were on suppressive ART (mean age [SD] = 53.2 [9.0]) completed the UCLA Loneliness Scale-Version 3 and the Center for Epidemiologic Studies Depression Scale as part of a comprehensive evaluation. Biomarkers of systemic inflammation (CRP, IL-6, CCL2/MCP-1, sCD14) and coagulation (D-dimer) were measured in blood using commercial immunoassays.

**Results:**

Multivariable linear regression analyses revealed that higher D-dimer, CCL2/MCP-1, and sCD14 were significant predictors of loneliness (*p*s < .05) while accounting for relevant covariates. Stepwise multiple linear regression models that included loneliness, biomarkers, and their interactions as predictors of depressive symptoms revealed significant main effects of loneliness and CCL2/MCP-1 levels (*p*s < .05), and a significant loneliness by D-dimer interaction (*p* < .05) whereby higher D-dimer was associated with increased depressive symptoms only at higher levels of loneliness.

**Conclusions:**

Increased coagulation activity is associated with loneliness, and in the context of loneliness, may increase risk for depression. Increased inflammation was associated with depression suggesting potentially dissociable underlying biological processes. To the extent that these processes are modifiable, such findings could have important implications in the treatment of loneliness and depression in PWH.

## Introduction

Loneliness, an aversive emotional experience tied to the discrepancy between one’s desired and experienced social relations (Perlman and Peplau 1981), is common among people with HIV (PWH) and is increasingly recognized as an important and understudied health determinant (Grov et al. 2010; Holt-Lunstad et al. 2015; Luo et al. 2012). Loneliness (or perceived social isolation) is distinct from objective social isolation (e.g., a lack of contact between an individual and society), or limited social interactions, in that it captures the extent to which an individual feels meaningfully connected to their social world (Hawkley and Cacioppo 2010). For example, some individuals may feel lonely despite having many social contacts and/or interactions; on the other hand, those who prefer to live solitary lives may not necessarily feel lonely. Studies in the general population have linked loneliness to a host of adverse medical (e.g., cardiovascular disease), psychiatric (e.g., depression, substance use), and neurocognitive (e.g., dementia) consequences (Luo et al. 2012). In older adults, loneliness has also been linked to elevated use of medications such as opioids and benzodiazepines (Vyas et al. 2021). Additionally, loneliness is a risk factor for mortality (Holt-Lunstad et al. 2015; Rico-Uribe et al. 2018) and is a major contributor to the escalating rates of suicides and opioid-related deaths that have led to a reduction in average lifespan in the US over the past two decades (Jeste et al. 2020).

Loneliness is more common in individuals with chronic illnesses relative to the general population (Pyle and Evans 2018). In addition to living with a chronic illness, PWH may be even more vulnerable to loneliness due to factors such as stigma, substance use, psychiatric disorders, and lower socioeconomic status (Grov et al. 2010). Studies on loneliness in PWH have found evidence of greater loneliness (Vance 2006) relative to people without HIV (PWoH). Prevalence estimates of loneliness in PWH vary across the literature depending on the method of assessment and/or specific operational definition of loneliness used (including different cutoffs on measures of loneliness), as well as differences in sample characteristics (e.g., age, race/ethnicity, psychiatric, and/or substance use characteristics). Studies generally suggest that about 36–58% of PWH report at least one symptom of loneliness (Greene 2018; Nachega et al. 2012; Rodkjaer et al. 2010). Rates of loneliness have been found to be higher in older PWH (≥ 50 years old; 46–64%) compared to younger (< 50 years) PWH (Mazonson et al. 2020; Yoo-Jeong et al. 2019). Greene (2018) evaluated the prevalence of loneliness in *n* = 356 PWH over age 50 using the UCLA 8-item loneliness scale (scores range from 8–32) and found that 58% reported at least one symptom of loneliness, with 24%, 22%, and 12% reporting mild (17–20), moderate (21–24), and severe (> 24) loneliness, respectively. In this study (Greene 2018), lonely PWH reported fewer relationships and were more likely to report depression and substance use (e.g., alcohol, tobacco). Common predictors of loneliness in PWH include social support, network size, and stigma/discrimination, which can be elevated among PWH (Harris et al. 2020). Although factors such as loneliness and social support are closely linked, and both are supported by cognitive theory that relies heavily on perceived, subjective evaluation of social networks (Zhang and Dong 2022), the biological processes between these factors appear to be distinct. For example, aspects of social engagement such as objective social isolation and social support have been associated with lower inflammation, such as lower CRP, while loneliness has been associated with poorer *regulation* of inflammatory processes, such as lower insulin like growth factor-1 (Walker et al. 2019). Importantly, loneliness has been linked to a range of negative health outcomes in PWH, including poorer sleep, less physical activity, increased stress, more risky sexual and drug use behaviors, increased risk of neurocognitive impairment, and poorer overall quality of life (Bryant et al. 2018; Fekete et al. 2018; Golub et al. 2010; Greene 2018; Han et al. 2017; Harris et al. 2020; Hussain et al. 2022; Mannes et al. 2016).

Large-scale studies in the general population across the adult lifespan have shown a significant association between loneliness and depression (Erzen and Cikrikci 2018). This relationship also has been demonstrated in PWH (Fekete et al. 2018; Grov et al. 2010; Rodkjaer et al. 2010). In one study of older PWH, increased loneliness explained an additional 8% of the variance in depression symptoms, after accounting for HIV-associated stigma, neurocognitive impairment, fatigue, and age (Grov et al. 2010). Longitudinal research examining the relationship between loneliness and depression in PWH is limited. Studies in the general population have suggested that there are likely bidirectional relationships between loneliness and depression, such that while loneliness often precedes onset of depression, loneliness and depression can exacerbate each other in a cyclical manner (Nuyen et al. 2020). The co-occurrence of loneliness and depression is also problematic and has been associated with increased sexual and drug risk-taking behaviors in PWH (Siconolfi et al. 2013; Su et al. 2018; Wang et al. 2017).

Despite its high prevalence and clinical consequences, little is known about biological processes associated with loneliness in PWH. Chronic inflammation, which persists even in the context of viral suppression on ART, has been linked to several adverse health outcomes in PWH (Deeks et al. 2013; Fukui et al. 2018; Nordell et al. 2014). The biopsychosocial relationships between inflammation and its consequences such as loneliness are complex. Inflammation can cause significant changes in social behavior (Eisenberger et al. 2017). For example, it is suggested that increased inflammation can lead to heightened sensitivity to negative, threatening social experiences (Moieni and Eisenberger 2018). Inflammation can also be a physiological response to loneliness. While acute loneliness may serve adaptive functions such as pursuit of meaningful social interactions, chronic loneliness may increase perception of social threat and hypervigilance that activates a chronic inflammatory response (Cacioppo et al. 2011), which can have adverse downstream health effects (Hawkley et al. 2003; Kiecolt-Glaser et al. 2010). Consistent with these notions, a recent review and meta-analyses conducted in individuals aged 16 or older from the general population found evidence supporting the link between inflammation and loneliness, though this publication noted limitations with regards to methodological heterogeneity across studies (Smith et al. 2020). Many of the available loneliness studies examined biomarkers of systemic inflammation that have been linked to downstream health-related consequences such as increased cardiovascular risk (Hawkley et al. 2010); these inflammatory processes include cytokines (e.g., interleukin-6 [IL-6]), chemokines (e.g., chemokine (C–C motif) ligand 2/monocyte chemoattractant protein-1 [CCL2/MCP-1]) and acute phase proteins (e.g., C-reactive protein [CRP]; Hackett et al. 2012; Leschak and Eisenberger 2019; Nersesian et al. 2018; Smith et al. 2020).

Pro-inflammatory and immune activation markers are elevated in PWH, and chronic inflammation is common even among PWH who are on suppressive ART. Research in PWH has also demonstrated links between inflammation and psychosocial disturbances. For example, Ellis et al. (2021) and Sun-Suslow et al. (2020) found that poorer social support was associated with higher inflammatory biomarkers in plasma and CSF in PWH. Literature in PWoH suggests that the link between social support and inflammation may be mediated through depressive symptoms (Santini et al. 2015). To our knowledge, only one study has examined the relationship between loneliness (specifically, as opposed to social isolation or social support) and inflammation in PWH. Derry et al. (2021) examined cross-sectional associations between psychosocial factors and inflammatory markers (IL-6, interferon-gamma [IFN-γ], tumor necrosis factor-alpha [TNF-α], and CRP) in older PWH (aged 54–78; n = 143), 93% of whom had a viral load < 200 copies/mL; the authors of this study did not find significant associations between loneliness and biomarker levels. Future research is needed to determine possible explanations for the discrepant findings between this study and research in PWoH. The authors suggested that the link between inflammation and loneliness could perhaps be stronger in younger PWH. Another possibility is that, in the context of chronic inflammatory conditions such as HIV, other unmeasured inflammation-related processes may be involved. For example, studies have found associations between greater coagulation activity (e.g., higher levels of fibrinogen and/or D-dimer) and poorer perceived social support in both PWoH (Wirtz et al. 2009) and PWH (Sun-Suslow et al. 2020). Since D-dimer is associated with similar health-related outcomes as loneliness such as cardiovascular disease (Leigh-Hunt et al. 2017; Valtorta et al. 2016), it is possible that coagulation activity may be another biological process related to loneliness. Thus, in addition to examining the relationship between biomarkers of inflammation and loneliness in PWH, the current study also sought to investigate the relationship between loneliness and D-dimer, a marker of coagulation activation and chronic inflammation, which is elevated and associated with adverse outcomes (e.g., cardiovascular disease, mortality) in PWH (Ford et al. 2010; Kuller et al. 2008).

Inflammation also has been implicated in the pathogenesis of depression in the general population (Kiecolt-Glaser et al. 2015; Miller et al. 2009), and in individuals at risk for HIV (Lu et al. 2019). In PWH, a number of studies have found associations between depression and biomarkers of immune activation and inflammation such as CRP, IL-6, and soluble cluster of differentiation-14 (sCD14; Lu et al. 2019; Norcini Pala et al. 2016; Rivera-Rivera et al. 2016; Stewart et al. 2020), even in PWH who are virally suppressed (Ellis et al. 2020). Moreover, inflammation-related vascular biomarkers also have been linked to depressive symptoms in PWH such as D-dimer (Norcini Pala et al. 2016; Stewart et al. 2020).

Despite the above referenced studies, there remains a limited understanding of the complex biopsychosocial relationships between loneliness and inflammation in PWH, as well as the complex biopsychosocial relationships between loneliness, inflammation, and depression. Our study sought to contribute knowledge to this area of research by examining: (1) the relationships between loneliness and biomarkers of systemic inflammation (CRP, IL-6, CCL2/MCP-1, sCD14) and related processes (e.g., coagulation; D-dimer), and (2) the combined influence of loneliness and inflammation on depressive symptoms.

## Methods

### Participants and Study Design

This cross-sectional study included 82 PWH from the NIMH-funded Multi-Dimensional Successful Aging among Adults living with HIV study, which was conducted at the University of California San Diego (UCSD) HIV Neurobehavioral Research Program (HNRP) and Sam and Rose Stein Institute for Research on Aging (SIRA). IRB approval was obtained from the UCSD Human Research Protections Program, and written consent was obtained from participants after they were informed of the design and purpose of the study, risk and benefits to participation, compensation, data sharing, confidentiality, and participant rights. Visits for the current study took place between November 2015 and December 2018.

### Inclusion/Exclusion Criteria

To enroll a representative cohort of participants, the parent study (i.e., the Multi-Dimensional Successful Aging Among Adults living with HIV study) applied minimal overall exclusion criteria. These were: 1) neurologic condition (not attributable to HIV) known to impact neurobehavioral functioning (e.g., Alzheimer's disease, stroke, traumatic brain injury), 2) psychotic disorders (e.g., schizophrenia), and 3) positive urine toxicology on the day of testing (except for cannabis). Inclusion criteria for the parent study were: 1) aged 36–65 years, 2) fluent in English, and 3) ability to provide informed consent. This study included all PWH from the parent study who completed the UCLA Loneliness Scale—Version 3 and the Center for Epidemiological Studies Depression Scale (CES-D), had complete biomarker data, were currently taking antiretroviral therapy (ART), and were virally suppressed (undetectable plasma HIV RNA viral load [< 50 copies/mL]).

### Assessments

#### Loneliness

Loneliness was measured using the revised UCLA Loneliness Scale – Version 3 (UCLA-3), a self-report measure that assesses the degree to which one perceives themselves to be socially disconnected or isolated from others (Russell 1996). The UCLA-3 is comprised of 20-items that required a Likert-type response from 1 (Never) to 4 (Always). Example items include, “How often do you feel that people are around you but not with you?” and “How often do you feel that your relationships with others are not meaningful?” Notably, no item explicitly uses the word “lonely.” The UCLA-3 revisions incorporated ten reverse-scored items and simplified the language from previous versions to make it more easily comprehensible to people of all education levels. After reverse-scoring, item responses are summed to provide a total score ranging from 20 to 80, with higher scores reflecting more loneliness. A cut off ≥ 44 on the UCLA-3 is indicative of high loneliness (Lee et al. 2019). This scale has previously been used to assess loneliness in PWH (Grov et al. 2010), and had excellent internal consistency in our sample (Cronbach’s *α* = 0.94).

#### Depressive Symptoms

Depressive symptoms were measured using the Center for Epidemiological Studies Depression Scale (CES-D). This 20-item self-report measure assesses the frequency of depressive symptoms, such as dysphoria, anhedonia, poor appetite, sleep disturbance, difficulty in thinking/concentration, worthlessness, fatigue, or suicidal ideation, that are experienced by the participant over the past week (Radloff 1977). Participants’ responses on each item can range from 0 to 3 (0=Rarely or none of the time [< 1 day in the past week]; 1=Some or a little of the time [1-2 days]; 2=Occasionally or a moderate amount of time [3-4 days]; 3=All of the time [5-7 days]). After reverse-scoring appropriate items, a total score is derived by summing item responses. The total score can range from 0 to 60, with higher scores reflecting a greater frequency of depressive symptoms, and a score of ≥16 suggestive of clinically significant depressive symptoms (Lewinsohn et al. 1997). The CES-D has good sensitivity and specificity in identifying Major Depressive Disorder, has been used extensively across many research and clinical populations including PWH (Chenneville et al. 2019; Lewinsohn et al. 1997; Marando et al. 2016), and showed good internal consistency in our sample (Cronbach’s *α* = 0.80).

#### Psychosocial Assessment

Participants were also administered a comprehensive neurobehavioral evaluation, which included collection of demographic data as well as psychiatric, substance use, and social characteristics. Psychiatric histories such as current and lifetime Major Depressive Disorder (MDD), as well as current and lifetime histories of substance use disorders, were assessed using the Composite International Diagnostic Interview (CIDI) version 2.1 (Kessler and Ustun 2004; Wittchen 1994), which follows *Diagnostic and Statistical Manual of Mental Disorders, Fourth Edition* (*DSM-IV*) criteria (APA 1994), consistent with other ongoing research studies at our center. The 4-item Duke Social Support Index (Koenig et al. 1993) and the MacArthur Health Aging questionnaire (Seeman et al. 1994) were administered to collect information regarding social interactions and perceived emotional support, respectively. Social interactions, a proxy for measuring objective social isolation, was indexed by summing two items from the Duke Social Support Index that ask participants to indicate how many times in the past week they either spent time with, or spoke on the phone with someone who did not live with them. Responses for each item were coded as 0 (“none”) to 7 (“seven or more times”; possible range of scores=0-14). Two items from the Macarthur Health Aging questionnaire were used as an index of perceived emotional support. These items asked participants to rate on a 4-point scale ranging from 1 (“Never”) to 4 (“Frequently") how often their spouse, children, close friends and/or relatives: 1) make them feel loved and cared for, and 2) are willing to listen when they need to talk about worries and problems. These two items were summed for an index of perceived emotional support (possible range of scores=0-8) and showed good internal consistency in our sample (Cronbach’s *α* = 0.83).

#### Neuromedical Assessment

The neuromedical assessment included a clinical interview to gather a comprehensive medical history (e.g., medical conditions, medications, etc.) as well as a blood draw, breathalyzer and urine drug screen, and self-report questionnaires (e.g., AIDS Clinical Trials Group [ACTG] Adherence Questionnaire [4-day adherence of > 90%]; Chesney et al. 2000). HIV status was determined by standard enzyme-linked immunosorbent assay (ELISA) and a confirmatory Western blot. HIV RNA was quantified in plasma by reverse transcriptase-polymerase chain reaction using a commercial assay with lower limit of quantification of 50 copies/mL. Hepatitis C Virus (HCV) serostatus was determined by commercial immunoassay.

Plasma levels of CRP, IL-6, CCL2/MCP-1, sCD14, and D-dimer were measured by the parent study using commercial enzyme-linked immunosorbent assay kits. Specifically, CRP was assayed by Lab Corp, IL-6 and CCL2/MCP-1 were run on a Mesoscale Discovery Imager 2400, and sCD14 and D-dimer were measured on a Molecular Devices ELISA microplate reader.

We considered each of these biomarkers of inflammation (CRP, IL-6, CCL2/MCP-1, sCD14; Kuller et al. 2008; Lau et al. 2006; Lien et al. 1998; Sevigny et al. 2007) and associated processes (e.g., coagulation; D-dimer; Freiberg et al. 2016; Kuller et al. 2008; Montoya et al. 2019) given their relevance to HIV disease. Additionally, these biomarkers are linked to psychosocial disturbances in PWoH such as social isolation and poor social support (Hackett et al. 2012; Leschak and Eisenberger 2019; Smith et al. 2020) and PWH (Ellis et al. 2021; Sun-Suslow et al. 2020), as well as their associations with loneliness in people without HIV (Cole et al. 2007).

#### Statistical Analyses

Variables of interest were checked for normality and skewness. Biomarkers were log-transformed to better improve fit, and outliers were removed if they were > 4 SDs from the mean. UCLA-3 total scores were normally distributed. CES-D total scores were skewed towards the lower range. However, these values were still acceptable based on absolute-value cutoffs for skewness and Kurtosis (3 and 10, respectively) that have been previously established by Kline (2011). Given that both outcome variables (loneliness and depressive symptoms) were not significantly skewed, multivariable regression were appropriate for use. Biomarkers were log-transformed given their significant skewness. CRP and D-dimer remained skewed after log transformations; therefore, non-parametric approaches (e.g., Spearman’s *ρ*) were utilized for bivariate analyses that included biomarkers. Cohen’s *d* was calculated to estimate effect size (Cohen 1988). The assumption of linearity was checked by examining linear and nonlinear relationships between each biomarker (CRP, IL-6, CCL2/MCP-1, sCD14, and D-dimer) and each dependent variable (loneliness symptoms and depressive symptoms) using bivariate and residual analyses (i.e., diagnostic residual plots such as Response Residual by Response Predicted Plots, Residual Normal Quantile Plots, Response Actual by Response Predicted Plots, Residual by Row Plots, and Residual by X Plots). There were no significant nonlinear associations between independent and dependent variables, and residual analyses confirmed linearity between these associations. Therefore, the assumption of linearity was satisfied in order to use multiple regression analysis. In addition, multicollinearity between our variables were checked first by analyzing the bivariate correlations between measures, then by calculating the variance inflation factor (VIF). Bivariate correlations between measures all fell below the threshold for multicollinearity (*r* = 0.7) suggested by Pallant (2020). Additionally, the VIF for all variables was less than 2.0, which was well below the suggested cutoff of 10 (Hair et al. 2014). Thus, multicollinearity did not exist in this study. All analyses were conducted with JMP Pro 16 statistical software.

First, Spearman’s rank-order correlation (*ρ*) tests were conducted to examine bivariate associations between biomarkers of systemic inflammation and coagulation (CRP, IL-6, sCD14, CCL2/MCP-1, and D-dimer) with loneliness (i.e., UCLA-3 total loneliness score) and depressive symptoms (i.e., CES-D Total Score). Two separate multivariable regressions were then conducted, one with loneliness as the outcome variable and the other with depressive symptoms as the outcome variable and loneliness as a predictor. All biomarkers were considered in multivariable analyses to examine their independent influences, after accounting for the relative variance predicted by other biomarkers on the outcome variables. Other candidate covariates from Table 1 (i.e., demographic, medical, psychiatric, social, and HIV disease characteristics) were included in multivariable regression models if they demonstrated bivariate trend-level associations (*p* < 0.10) with the outcome variable (i.e., loneliness or depressive symptoms). Age was considered *a* priori for inclusion in multivariable analyses predicting loneliness given its association with loneliness in the literature (Dyal and Valente 2015) and associations with systemic inflammatory biomarkers (Fukui et al. 2018). Interactions between loneliness and biomarkers of systemic inflammation were also included in the second model predicting depressive symptoms to evaluate whether loneliness may moderate the relationship between inflammation and depression in PWH. Backwards stepwise regression was conducted for each outcome and a minimal Akaike information criterion (AIC; Akaike 1974) was used to reduce the model. The AIC method was preferred over the coefficient-based stepwise regression because AIC is a more data-driven approach, which balances goodness of fit and model complexity while the coefficient-based approach relies more on theoretical framework for the regression model. Given that loneliness, inflammation, and depression literature in PWH is still limited and somewhat inconsistent, AIC was chosen as the appropriate method to use for our more exploratory aims.

**Table 1:** Participant Characteristics

	PWH (*N* = 82)
Demographics	
Age (years)	53.2 (9.0)
Education (years)	14.5 (2.8)
Sex/gender (% male)	86.6%
Race/ethnicity	–
White, non-Hispanic (%)	58.5%
Hispanic (%)	19.5%
Black (%)	13.4%
Other^a^ (%)	8.5%
HIV Disease Characteristics	
Estimated duration of HIV disease (years)	16.3 [4.0, 25.5]
AIDS Status (%)	58.5%
Nadir CD4 count	188.5 [53.8, 353.0]
Current CD4 + T-cell count	678.0 [524.5, 911.0]
Taking ART (% currently on ART)	100.0%
Duration of current ART regimen (years)	1.1 [0.4, 2.4]
Plasma HIV viral load (% undetectable^b^)	100.0%
Psychiatric Diagnoses	
Current Major Depressive Disorder (% yes)	7.4%
Lifetime Major Depressive Disorder (% yes)	50.0%
Current Substance Abuse Diagnosis (% yes)	7.4%
Lifetime Substance Use Disorder^c^ (% yes)	59.8%
Lifetime Alcohol Use Disorder (% yes)	40.2%
Lifetime Cannabis Use Disorder (% yes)	24.4%
Lifetime Other^d^ Substance Use Disorder (% yes)	43.9%
Medical Comorbidities	
Obesity (% Body Mass Index ≥ 30)	28.4%
Hepatitis C Virus (% yes)	15.9%
Diabetes (% yes)	12.2%
Hypertension (% yes)	46.3%
Hyperlipidemia (% yes)	43.9%
Social Resources	
Social Interactions (# in past week)^e^	6.0 [3.0, 9.3]
Perceived Emotional Support (Total)^f^	3.4 (0.9)
Perceived Emotional Support (% Frequent Support)	54.9%
Primary Study Variables	
Loneliness Total (UCLA-3^g^ total score)	40.6 (12.0)
Loneliness Elevated^h^ (%)	34.2%
Depressive Symptoms Total (CES-D^i^ total score)	19.4 (8.3)
Depressive Symptoms Elevated^j^ (%)	57.3%
Plasma Biomarkers	
Log10(CRP)	0.08 [-0.22, 0.41]
Log10(D-dimer)	2.67 [2.58, 2.82]
Log10(IL-6)	-0.16 [-0.05, -0.28]
Log10(CCL2/MCP-1)	2.26 [2.22, 2.32]
Log10(sCD14)	6.06 [5.75, 6.18]

Mean (SD) or Median [IQR];

^a^Race/ethnicity other than White, Black, or Hispanic; ^b^plasma HIV RNA viral load ≤ 50 copies/mL; ^c^Substance Use Disorder = DSM-IV lifetime substance abuse and/or dependence diagnosis; ^d^Other = illicit substances of abuse (most prevalent = methamphetamine, cocaine, and opioid); ^e^Total number of social interactions in the past week determined using items from the 4-item Duke Social Support Index; ^f^Frequency of perceived emotional support using items from the MacArthur Healthy Aging Questionnaire; ^g^Revised UCLA Loneliness Scale – Version 3 (UCLA-3); ^h^UCLA-3 ≥ 44; ^i^Center for Epidemiological Studies Depression Scale (CES-D); ^j^CES-D ≥ 16; CRP = C-reactive protein; IL-6 = Interleukin-6; CCL2/MCP-1 = Chemokine (C–C motif) ligand 2/ monocyte chemoattractant protein-1; sCD14 = soluble cluster of differentiation 14

## Results

### Participant Characteristics

Participant characteristics are presented in Table 1. The study sample was 87% male and 59% non-Hispanic White with a mean age of 53.2 years (range: 36–69 years) and mean education of 14.5 years. Participants’ median estimated duration of HIV disease was 16.3 years (interquartile range [IQR] = [4.0, 25.5]). All participants were currently on ART and were virally suppressed (plasma HIV viral load ≤ 50 copies/mL). Median nadir and current CD4^+^ T-cell counts were 188.5 (IQR = [53.8, 353.0]) and 678.0 (IQR = [524.5, 911.0]), respectively. About 60% of our sample met criteria for lifetime substance use disorder with alcohol use disorder being the most prevalent (40.2%). Table 1 provides additional participant characteristics such as current and lifetime psychiatric and substance use histories, common comorbid medical conditions, social interaction and support characteristics, total loneliness scores (i.e., Total UCLA-3 Score), depressive symptoms (i.e., Total CES-D Score), and distributions of biomarkers in our sample. Approximately 34.2% and 57.3% reported elevated loneliness and depression, respectively.

### Bivariate and multivariable analyses examining associations between loneliness and inflammation

At the bivariate level, loneliness was significantly associated with higher levels of D-dimer (Spearman’s *ρ* = 0.26; *p* = 0.02), but not with the remaining biomarkers (CRP, IL-6, CCL2/MCP-1, and sCD14; Spearman’s *ρ*s range 0.06–0.14; *p*s > 0.10). Table 2 displays the predictors identified as candidates for multivariable modelling. Predictors included all biomarkers (rationale stated above), age given its associations with loneliness and biomarkers in the literature, and variables from Table 1 that were associated (*p* < 0.10) with loneliness at the bivariate level (i.e., race/ethnicity, social interactions and perceived emotional support, nadir CD4^+^, and lifetime illicit [i.e., other than alcohol or cannabis] substance use disorder). The final model selected by AIC accounted for 49.3% of the variance in loneliness (*F*(7,74) = 10.3, *p* < 0.0001; See Table 2). D-dimer, sCD14, and CCL2/MCP-1 were significant independent predictors of loneliness in the multivariable model, which also included less frequent emotional support (*B* = 0.45, 95% CI = [0.38, 1.62], *p* < 0.001), higher nadir CD^+^ T-cell count (*B* = 0.35, 95% CI = [0.10, 1.87] *p* < 0.001), and presence of lifetime substance use disorder (*B* = 0.24, 95% CI = [0.036, 2.68], *p* = 0.007). Younger age was associated with loneliness at the trend level (*B* = -0.24, 95% CI = [-0.36, 0.41], *p* = 0.083). To ensure these findings were not better explained by vascular risk factors (e.g., smoking, hypertension, obesity), which are common in PWH and associated with inflammation and coagulation in the literature, we added these variables to the model but their addition did not weaken the association between the significant biomarkers (CCL2/MCP-1, sCD14, and D-dimer) and loneliness.

**Table 2:** Summary of bivariate and multivariable analyses when loneliness is the outcome (as measured by UCLA-3 Total scores)

**Variable**	**Risk Direction** **(Lonelier)**	**Bivariate** **Analyses**			**Multivariable Analyses:** **Final AIC Model**		
		**Statistic**	**value**	***p*-value**	***B***	**95% CI**	***p*-value**
CRP^a^	–	*ρ*	0.12	0.278	–	–	–
IL-6^a^	–	*ρ*	0.10	0.355	–	–	–
CCL2/MCP-1^a^	–	*ρ*	0.06	0.568	**0.20**	**[0.11, 2.56]**	**0.029**
sCD14^a^	–	*ρ*	0.14	0.211	**0.19**	**[0.09, 1.84]**	**0.033**
D-dimer^a^	**Higher**	***ρ***	**0.26**	**0.017**	**0.32**	**[0.21, 2.0]**	** < 0.001**
Age	Younger	*ρ*	-0.12	0.294	-0.24	[-0.36, 0.41]	0.083
Social Interactions	**Fewer**	***ρ***	**0.26**	**0.016**	**–**	**–**	**–**
Emotional Support	**Less often**	***t***	**4.25**	** < 0.001**	**0.45**	**[0.38, 1.62]**	** < 0.001**
Race/ethnicity							
White, non-Hispanic	–	*t*	0.14	0.887	–	–	–
Hispanic	**Non-Hispanic**	***t***	**2.24**	**0.028**	–	–	–
Black	–	*t*	0.82	0.416	–	–	–
Other^b^	**Other**^b^	***t***	**2.43**	**0.018**	–	–	–
Nadir CD4^+^ T-cell count^c^	**Higher**	***ρ***	**0.30**	**0.005**	**0.35**	**[0.10, 1.87]**	** < 0.001**
Lifetime substance use disorder^d^	**Present**	***t***	**2.15**	**0.034**	**0.24**	**[0.06, 2.68]**	**0.007**
**Model Statistics**							
**F-ratio**					**10.3**		
***df***					**(7,74)**		
***R***^**2**^					**0.49**		
***p*-value**					** < 0.001**		

In multivariable analyses, the final model was established using backwards stepwise regression using the Akaike information criterion (AIC); UCLA-3 = UCLA Loneliness Scale-Version 3; CRP = C-Reactive Protein; IL-6 = Interleukin-6; CCL2/MCP-1 = Chemokine (C–C motif) ligand 2/monocyte chemoattractant protein-1; sCD14 = soluble cluster of differentiation 14

^a^log transformed; ^b^Self-reported race/ethnicity as other than White, Black, or Hispanic (e.g., more than one race); ^c^square root transformed; ^d^Lifetime abuse or dependence on substances other than alcohol or cannabis

### Bivariate and multivariable analyses examining the relationships between loneliness, inflammation, and depressive symptoms

**Fig. 1:**
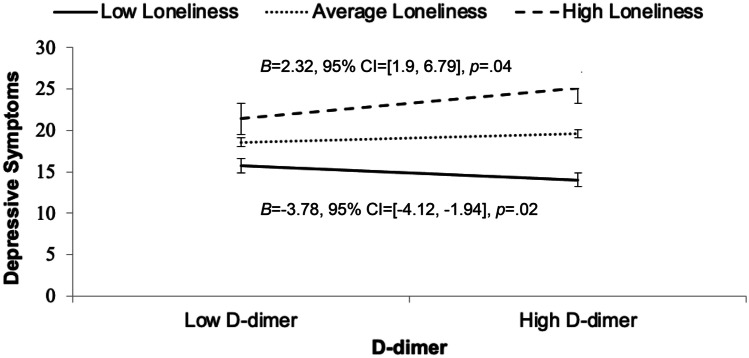
Higher loneliness scores (UCLA-3 Total scores), in conjunction with high D-dimer values, contributed to an additive effect on depressive symptoms (CES-D Total scores) in PWH

At the bivariate level, increased frequency of depressive symptoms (i.e., higher Total CES-D scores) was associated with greater loneliness (*ρ* = 0.53; *p* < 0.0001), as well as with higher levels of IL-6 (*ρ* = 0.23; *p* = 0.04) and CCL2/MCP-1 (*ρ* = 0.24; *p* = 0.03). To examine biomarkers that impact depression in the context of loneliness, and to test whether loneliness moderates the relationship between depressive symptoms and inflammation, a second stepwise regression was conducted. Table 3 displays the predictors identified as candidates for multivariable modelling, including loneliness, all biomarkers, and variables from Table 1 that were associated (*p* < 0.10) with depressive symptoms at the bivariate level (i.e., race/ethnicity, current CD4^+^ T-cell count, lifetime substance use disorder, and hypertension). The final model selected by AIC accounted for 54.4% of the variance in depressive symptoms (*F*(8, 73) = 10.9, *p* < 0.0001; See Table 3). Loneliness (*B* = 0.31, 95% CI = [0.19, 0.43], *p* < 0.0001) and higher CCL2/MCP-1 (*B* = 25.53, 95% CI = [11.58, 39.48], *p* = 0.0005) emerged as significant independent predictors of depressive symptoms. A significant interaction of loneliness by D-dimer was also found (*B* = 0.59, 95% CI = [0.01, 1.17], *p* = 0.0497) whereby a positive relationship between D-dimer and increased frequency of depressive symptoms only emerged at higher levels of loneliness. This finding is depicted graphically in Fig. 1. Race/ethnicity was the only other covariate associated with depressive symptoms (Black race/ethnicity was associated with a higher frequency of depressive symptoms, while Hispanic race/ethnicity and those identifying with a race/ethnicity other than White, Hispanic, or Black was associated with less frequent depressive symptoms).

**Table 3:** Summary of bivariate and multivariable analyses when depression is the outcome (as measured by CES-D Total Scores)

**Variable**	**Risk Direction** **(More depression)**	**Bivariate** **Analyses**			**Multivariable Analyses:** **Final AIC Model**		
		**Statistic**	**value**	***p*-value**	***B***	**95% CI**	***p*-value**
Loneliness (UCLA-3 Total)	**Lonelier**	***ρ***	**0.53**	** < .0001**	**0.31**	**[0.19, 0.43]**	** < 0.0001**
CRP^a^	–	*ρ*	0.10	0.36	–	–	–
IL-6^a^	**Higher**	***ρ***	**0.23**	**0.04**	3.59	[-3.07, 10.24]	0.29
CCL2/MCP-1^a^	**Higher**	***ρ***	**0.24**	**0.03**	**25.53**	**[11.58, 39.48]**	**0.0005**
sCD14^a^	–	*ρ*	0.03	0.76	–	–	–
D-dimer^a^	–	*ρ*	0.15	0.17	3.39	[-4.58, 11.35]	0.40
Race/ethnicity							
White, non-Hispanic	–	*t*	0.51	0.61	–	–	–
Hispanic	**Non-Hispanic**	***t***	**3.25**	**0.002**	**-3.40**	**[-6.24, -0.55]**	**.02**
Black	**Black**	***t***	**3.28**	**0.002**	**8.06**	**[4.88, 11.24]**	** < .0001**
Other^b^	–	*t*	0.33	0.75	**-5.35**	**[-9.12, -1.58]**	**.006**
Current CD4^+^ T-cell count^c^	**Higher**	***ρ***	**0.23**	**0.04**	–	–	–
Lifetime substance use disorder^d^	–	*t*	1.96	0.05	–	–	–
Hypertension	**Present**	***t***	**2.01**	**0.047**	–	–	–
Loneliness x D-dimer	–	*–*	–	–	**0.59**	**[0.01, 1.17]**	**.0497**
**Model Statistics**							
**F-ratio**					**10.9**		
***df***					**(8,73)**		
***R***^**2**^					**0.54**		
***p*-value**					** < 0.001**		

In multivariable analyses, the final model was established using backwards stepwise regression using the Akaike information criterion (AIC); UCLA-3 = UCLA Loneliness Scale-Version 3; CES-D = Center for Epidemiologic Studies Depression Scale; CRP = C-Reactive Protein; IL-6 = Interleukin-6, CCL2/MCP-1 = Chemokine (C–C motif) ligand 2/monocyte chemoattractant protein-1; sCD14 = soluble cluster of differentiation 14

^a^log transformed; ^b^Self-reported race/ethnicity as other than White, Black, or Hispanic (e.g., more than one race); ^c^square root transformed; ^d^Lifetime abuse or dependence on substances other than alcohol or cannabis

To ensure these relationships were not better explained by factors associated with inflammation and/or coagulation (e.g., age, acute/early HIV infection, or vascular risk factors such as hyperlipidemia and smoking status), these additional variables were considered alongside age, ethnicity, lifetime substance use disorder, loneliness, and biomarkers of inflammation in a follow-up stepwise regression analysis. Despite accounting for these risk factors, results remained unchanged.

## Discussion

Loneliness is common among PWH and is associated with depression and other adverse health-related consequences, including non-AIDS-associated comorbidities (e.g., cardiovascular disease; CVD) and increased risk of mortality. However, the biological processes associated with loneliness in PWH are not well understood. Moreover, the biopsychosocial relationships between loneliness, inflammation, and depression in PWH are complex, and research examining these relationships simultaneously in PWH is sparse. Our results suggest that loneliness is associated with increased coagulation activity (D-dimer) and monocyte activation (sCD14, CCL2/MCP-1) in virally suppressed PWH while accounting for demographics (e.g., age, race/ethnicity), and relevant psychosocial covariates (e.g., social interactions, perceived emotional support). Loneliness was, as expected, associated with depressive symptoms, as were higher levels of CCL2/MCP-1, though D-dimer was only associated with more frequent depressive symptoms at higher levels of loneliness. These findings provide insight into the potential biological processes associated with loneliness in virally suppressed PWH that may be, to some extent, dissociable from those of depressive symptoms. This knowledge may help improve treatment efforts aimed at remediating these highly prevalent conditions and reducing their impact on health-related outcomes in PWH.

Regarding our first aim, to our knowledge, this study is the first to report significant associations between loneliness and biomarkers of systemic inflammation (sCD14, CCL2/MCP-1) and coagulation (D-dimer) in ART-treated and virally suppressed PWH, providing valuable insight into the biological mechanisms by which loneliness may impact health in this population. Increased coagulation activity (as indexed by higher levels of D-dimer) was associated with loneliness in both unadjusted (i.e., bivariate correlations) and adjusted analyses (i.e., stepwise backwards AIC multivariable regression), and biomarkers of monocyte activation and microbial translocation (CCL2/MCP-1, sCD14) were associated with loneliness in adjusted models. Other significant predictors of loneliness included lower emotional support, higher nadir CD4^+^ T-cell count, and lifetime substance use disorder. Importantly, these results appear to be independent of relevant comorbidities since we adjusted final models for common cardiovascular risk factors (e.g., smoking status, hypertension, hypercholesterolemia, diabetes) and hepatitis C virus to ensure our findings were not better explained by these factors.

D-dimer is a fibrin degradation product that reflects ongoing activation of the coagulation system. Higher levels of D-dimer are indicative of coagulation imbalance, which can be both a cause and amplifier of the inflammatory response (Funderburg and Lederman 2014). CCL2/MCP-1 is a chemokine produced constitutively or in response to proinflammatory stimuli that is involved in the recruitment of monocytes, lymphocytes, and other cells to sites of infection and inflammation peripherally and in the CNS (Gu et al. 1997). Soluble CD14 is released from monocytes and macrophages following stimulation by proinflammatory cytokines, bacterial lipopolysaccharide (LPS), and other ligands (Shive et al. 2015). Previous studies have linked loneliness to elevated levels of CCL2/MCP-1 in PWoH (Hackett et al. 2012, 2019), though to our knowledge no studies have examined associations between loneliness and D-dimer or sCD14. Nersesian et al. (2018) found an association between levels of fibrinogen, which, with D-dimer can lead to a hypercoagulable state, and loneliness in PWoH. Associations between loneliness and social support, as well as with comorbid substance use disorders, has been reliably demonstrated in the literature (Greene et al. 2018; Stanton et al. 2015). In PWH, studies have linked poorer social support, which is related to, but independent of loneliness, with CCL2/MCP-1 in PWH (Ellis et al. 2020), as well as with D-dimer in PWH (Sun-Suslow et al. 2020), and with D-dimer and fibrinogen levels after acute psychosocial stress (Wirtz et al. 2009). Our findings extend these results to demonstrate effects of systemic inflammation on loneliness independent of social support, though additional studies are needed to further examine these interrelationships. Importantly, higher levels of D-dimer, CCL2/MCP-1, and sCD14 are observed even in treated and virally suppressed PWH (Mendez-Lagares et al. 2013) and have been linked to CVD (Joven et al. 2006; Kelesidis et al. 2012; Longenecker et al. 2014; Zungsontiporn et al. 2016) and increased risk for mortality (Duprez et al. 2012).

As expected, loneliness was associated with higher frequency of depressive symptoms in both adjusted and unadjusted analyses, which is consistent with existing literature in PWH (Grov et al. 2010) and PWoH (Lee et al. 2019). The robustness and strength of the loneliness main effect in explaining variance in depressive symptoms above and beyond inflammation and immune health was notable given the extensive evidence supporting the role of inflammation in depression (Osimo et al. 2020). Higher levels of monocyte activation marker CCL2/MCP-1 also were independently associated with increased depressive symptoms at the bivariate and multivariable levels, highlighting CCL2/MCP-1 as a biomarker sensitive of depressed mood independent of loneliness. This finding is consistent with studies that have observed higher CCL2/MCP-1 levels in individuals with major depressive disorder (Rajagopalan et al. 2001), as well as in individuals reporting even mild depressive symptoms (Suarez et al. 2003). Novel findings in the current study also demonstrated that the relationship between D-dimer and depressive symptoms was only present at higher levels of loneliness. This requires replication but provides further support for the specificity of the association between coagulation and loneliness, and suggests that if both are elevated, this could lead to combined adverse effects on depressive symptoms. Race/ethnicity was another significant predictor of increased depressive symptoms, whereby PWH who self-identified as Black reported greater depressive symptoms relative to those who self-reported as non-Black race/ethnicities. This race/ethnicity effect may reflect the impact of lifetime(s) of racial discrimination and systemic socioeconomic disadvantages, which were not directly measured in the current study, on increased risk for depression. Finally, higher nadir CD^+^ T-cell count was independently associated with loneliness, which was unexpected based on prior literature (Brouillette et al. 2022). Perhaps those with higher nadir CD^+^ T-cell counts represent an HIV survivor bias of those who may have experienced more HIV stigma/discrimination, thereby contributing to more feelings of loneliness. Future studies should investigate the links between elevated psychosocial disturbance with higher nadir CD^+^ T-cell count in more detail.

Our overall findings suggest that activation of inflammatory and/or coagulation pathways may be mechanisms associated with loneliness and depression within virally suppressed PWH. Feelings of loneliness can be both a cause of inflammation (e.g., via dysregulation of neuroendocrine and immune pathways) and a consequence of inflammation. For example, loneliness can be conceptualized as a threat to our social well-being, which activates the body’s stress-response (Hawkley and Cacioppo 2003). In the general population, chronic loneliness and subsequent heightened sensitivity to social threats have been shown to activate similar neuroendocrine pathways involved in chronic stress, releasing epinephrine, cortisol, and glucocorticoids, which can hinder immune function and amplify peripheral and central inflammation (Hackett et al. 2012). Research in healthy adults and clinical populations have demonstrated that lonelier people are more psychologically reactive to stress than those who are less lonely, and their responses to acute laboratory stressors are characterized by enhanced concentrations of inflammatory biomarkers, including CCL2/MCP-1 (Hackett et al. 2012, 2019). In addition, reliable increases in D-dimer levels have been observed in response to acute psychosocial stressors across clinical populations (Wirtz et al. 2006, 2008), highlighting the role of enhanced stress response in both inflammatory and coagulation pathways that may result from loneliness. Loneliness is also associated with difficulties in cognitive-behavioral processing such as a tendency to engage in negative appraisals (i.e., perceived poorer emotional support) and poorer health behaviors (i.e., substance use disorders), which may have downstream consequences on vascular and immune regulation (Cacioppo et al. 2000). Moreover, an association between stress-induced D-dimer levels and depression has been observed in PWoH (von Känel et al. 2008), which could help explain the relevance of loneliness in the relationship between D-dimer and depressive symptoms in our study. Given evidence that stress causes changes in gut microbiota and may play a role in gut dysbiosis (Mackos et al. 2017), it also is possible that psychosocial stress associated with loneliness may have similar effects that may explain, in part, the association we observed between elevated sCD14 and loneliness. In addition to enhanced inflammatory responses, loneliness is associated with reduced immune function and antiviral immunity (Jaremka et al. 2013; Leschak and Eisenberger 2019; Steptoe et al. 2004). Indeed, people who are lonely have been shown to be more susceptible to viral infections such as the common cold (LeRoy et al. 2017), and are more vulnerable to chronic diseases such as metabolic syndrome (Henriksen et al. 2019), heart disease (Valtorta et al. 2016) and even all-cause mortality risk (Holt-Lunstad et al. 2015). The dysregulation of immune and inflammatory response systems resulting from loneliness can have further downstream consequences on D-dimer, a measure of coagulation imbalance and a proxy for systemic inflammation within the body.

As noted above, multiple factors contribute to activation of inflammatory and coagulation pathways in PWH such as ongoing viral replication and co-infections (Deeks et al. 2013), which may help explain our loneliness and biomarker findings. Chronic immune activation also is associated with microbial translocation in both acute and chronic HIV infection (Brenchley et al. 2006; Somsouk et al. 2015) and in treated PWH (Cassol et al. 2010). In virally suppressed PWH, chronic monocyte activation and damage to the gut mucosa and associated microbial translocation are considered prominent drivers of persistent inflammation (Deeks et al. 2013; Godfrey et al. 2019). Microbial translocation causes an increase in circulating microbial products such as lipopolysaccharide (LPS) which activates monocytes leading to the production of sCD14 (Sandler and Douek 2012). Elevated levels of sCD14 and LPS can persist despite ART (Mendez-Lagares et al. 2013; Villanueva-Millan et al. 2017) and both have been associated with subclinical atherosclerosis (Longenecker et al. 2014). LPS-stimulated monocyte activation also leads to increased tissue factor expression which activates the coagulation cascade on the surface of monocytes (Funderburg et al. 2010). As a result, HIV causes direct and indirect activation of both the adaptive and innate immune systems, leading to a state of chronic low-level inflammation that persists in PWH despite viral suppression on ART. Over time, the combined persistent, chronic low-level inflammation may lead to other consequences including vascular dysfunction and coagulation imbalance, as well as atherosclerosis, CVD and other adverse health-related outcomes. In PWH, increased sCD14 and elevated tissue factor expression have been associated with D-dimer levels in PWH (Funderburg et al. 2010). Thus, there may be overlapping etiologies contributing to the associations observed in this study. Future research is needed to explore these bidirectional links and between loneliness and inflammation in PWH and the underlying biological pathways.

To our knowledge, only one other study has examined associations between inflammation and loneliness in PWH (Derry et al. 2021). This study also investigated associations between inflammation and depressive symptoms, though in separate analyses. They did not find significant associations between loneliness and inflammatory biomarkers in their sample of older PWH (aged 54–78). Interestingly, the only overlap in biomarkers investigated between our study and Derry et al. (2021) were IL-6 and CRP, neither of which reached significance in bivariate or multivariable analyses in our study as well. Some studies in PWoH have found positive associations between these biomarkers and loneliness, though the evidence is mixed (Smith et al. 2020). One possible explanation for the lack of associations in the current study, and in Derry et al. (2021), is that IL-6 and CRP may not be as sensitive to loneliness in the context of viral suppression. All or most participants in our studies were virally suppressed (100% ≤ 50 copies/mL in the current study and 93% < 200 copies/mL in Derry et al. (2021)). Another possibility is that the associations between some inflammatory processes and loneliness may be stronger in women, a finding that has been demonstrated in the literature in PWoH (Hackett et al. 2012). Given the limited number of females in our study (*n* = 11), we were unable to examine this possibility, but it should be considered for future studies.

By contrast, higher IL-6 levels were associated with *depressive* symptoms in PWH, which is consistent with other studies in PWH (Derry et al. 2021; Ellis et al. 2020) and PWoH (Yuan et al. 2019), though in our study this association did not remain significant in multivariable modelling. One possibility is that this association was influenced more strongly by other factors, such as race/ethnicity, which was also a significant predictor of depressive symptoms (i.e., more frequent depressive symptoms reported by Black PWH relative to other race/ethnicities). Post-hoc bivariate associations conducted to explore this possibility provided preliminary support for an association between higher IL-6 levels and depressive symptoms in Black PWH (*ρ* = 0.58, *p* = 0.059) that was not observed in the combined non-Black race/ethnicity groups (*ρ* = 0.14, *p* > 0.10). While our sample size of Black PWH in this study is quite small (*n* = 11), this possible association is consistent with findings linking IL-6 to depressive symptoms in a sample of PWH where 50% of participants self-reported as Black (Derry et al. 2021). Future studies are needed to further determine race/ethnicity-specific associations between systemic inflammation and depressive symptoms in virally suppressed PWH. We also did not find associations between CRP and depressive symptoms, which runs contrary to other studies in PWH (Ellis et al. 2020), though the evidence is mixed and other studies have not observed significant associations (Derry et al. 2021; Irwin et al. 2018). Additionally, Poudel-Tandukar et al. (2014) found an association between CRP and depressive symptoms only in PWH with higher levels of inflammation (CRP > 3 mg/L). Using this threshold, 21.4% of our sample (*n* = 19) had high CRP levels, although post hoc analyses did not reveal any significant associations between the dichotomous CRP variable and either loneliness or depressive symptoms (Cohen’s *d*s = 0.38 and 0.30, *p*s > 0.10). As with loneliness, other research suggests that the relationship between CRP and depression may be influenced by race/ethnicity and sex (Morris et al. 2011), which as mentioned above would be an important avenue for future research.

There are limitations to note regarding this study. First, our data were cross-sectional, which precludes making determinations regarding the directionality of associations. As noted in the introduction, there may be bidirectional relationships between loneliness, systemic inflammation, and depression that were unable to be fully examined given the cross-sectional nature of our study. Therefore, longitudinal follow-up studies are necessary to further elucidate the directionality of these findings. Second, our findings were derived from a relatively small sample size, and our sample was mostly male with approximately 58% being White and middle-to-older adults. In the future, efforts to expand the subject pool to include women (in order to better understand potential sex differences on loneliness, biomarkers, and depression), those across different age ranges (given that loneliness may be experienced differently across the lifespan including in critical periods of young adulthood and higher ends of older age [i.e., > 85 years]), and racially/ethnically underrepresented participants (who may experience more systemic challenges than others) are important to better improve generalizability of findings. Furthermore, our study lacked a comparison group of PWoH. However, the primary aim of this investigation was to examine inflammation as a mechanism underlying loneliness in HIV given that very limited work has been published to-date filling this gap in literature. By contrast, the links between loneliness and inflammation in people without HIV (PWoH) have been much more extensively studied. Also, the biomarker values in our PWH sample were comparable to other values in other published studies (Sun-Suslow et al. 2020) that had a PWoH comparison group, suggesting that our results are consistent and reliable despite the lack of control group. Additionally, biomarkers in this present study were selected based on their association with HIV-disease processes and prior reports of relationships with psychosocial outcomes, but by no means are all-inclusive. Is it possible that other biomarkers (e.g., microbiome, EEG, cardiovascular injury, fMRI) not examined in this study may have associations with loneliness and/or depression, which further highlights the need to continue growing this area of research. Finally, HIV-specific variables such as HIV-related stigma and discrimination, which has been associated with elevated loneliness in PWH, was not examined in the current study and would be important to include in future investigations to further explain HIV and loneliness findings (e.g., the positive relationship between loneliness and nadir CD + T-cell count observed in our study).

Despite these limitations, this study provides unique contributions to the literature that could have significant clinical implications. To our knowledge, this study is the first to demonstrate associations between loneliness and systemic inflammation and coagulation in PWH, and to show that there were both independent and combined influences of these factors on depressive symptoms. Ongoing research examining the efficacy of interventions for loneliness across many populations is promising, including among both younger and older adults without HIV, and in people with psychotic disorders such as schizophrenia (Masi et al. 2011; Shah et al. 2019, 2021). Given the compounding effect of loneliness and systemic inflammation on depressive symptoms, it may be possible to simultaneously target loneliness through social and cognitive-behavioral interventions, as well as by screening for and reducing elevated levels of loneliness and inflammation, when treating depression in PWH. Pharmacological interventions targeting the reduction of systemic inflammation and/or coagulation (e.g., anti-inflammatory medications, probiotics; Gori et al. 2011; Villar-Garcia et al. 2015) may be useful as potentially independent treatments for loneliness and/or depression, or as adjuncts to non-pharmacological interventions.

## Data Availability

Data cannot be shared publicly to maintain full participant confidentiality as there is a substantial risk of reidentification of study participants. Data were obtained from a specific and vulnerable population in a specific city in the USA that could become identifying despite efforts to anonymize data. However, data are available from the HIV Neurobehavioral Research Center’s Data Management and Information System (DMIS) Committee (contact: hnrpresource@ucsd.edu) to researchers who meet the criteria for access to confidential deidentified data.
